# Long-term benefits of physical activity in adult patients with late onset Pompe disease: a retrospective cohort study with 10 years of follow-up

**DOI:** 10.1186/s13023-023-02924-x

**Published:** 2023-10-11

**Authors:** Gamida Ismailova, Margreet A. E. M. Wagenmakers, Esther Brusse, Ans T. van der Ploeg, Marein M. Favejee, Nadine A. M. E. van der Beek, Linda E. M. van den Berg

**Affiliations:** 1 Department of Pediatrics, Center for Lysosomal and Metabolic Diseases, Erasmus Medical Center, Sophia Children’s Hospital, Mailbox 2060, 3000 CB Rotterdam, The Netherlands; 2 Department of Internal Medicine, Center for Lysosomal and Metabolic Diseases, Erasmus Medical Center, Mailbox 2040, 3000 CA Rotterdam, The Netherlands; 3 Department of Neurology, Center for Lysosomal and Metabolic Diseases, Erasmus Medical Center, Mailbox 2040, 3000 CA Rotterdam, The Netherlands; 4 Department of Physical Therapy, Erasmus Medical Center, Mailbox 2040, 3000 CA Rotterdam, The Netherlands; 5 Department of Orthopedics and Sports Medicine, Center for Lysosomal and Metabolic Diseases, Erasmus Medical Center, Sophia Children’s Hospital, Mailbox 2060, 3000 CB Rotterdam, The Netherlands

**Keywords:** Late onset Pompe disease, Exercise training program, WHO physical activity norm, Endurance, Muscle strength, Muscle function

## Abstract

**Background:**

In 2011 a 12 weeks personalized exercise training program in 23 mildly affected adult late onset Pompe patients (age 19.6–70.5 years) improved endurance, muscle strength and function. Data on long-term effects of this program or of other physical activity in Pompe disease are absent. This retrospective cohort study aimed to explore effects of long-term healthy physical activity according to the WHO norm and the former exercise training program on the disease course.

**Results:**

A total of 29 adult late onset Pompe patients were included: 19 former exercise training program participants and 10 comparable control patients. Patients, who based on interviews, met the 2010 WHO healthy physical activity norm (active, n = 16) performed better on endurance (maximal cardiopulmonary exercise test), muscle strength and function compared to patients not meeting this norm (inactive, n = 13) (*p* < 0.05). Majority of the outcomes, including endurance and manually tested muscle strength, tended to be higher in the active patients of the 2011 training cohort who continued the program compared to active control patients (*p* > 0.05).

**Conclusion:**

In Pompe disease long-term healthy physical activity according to the 2010 WHO norm leads to physical benefits and a personalized exercise training program may have additional favorable effects and both should be recommended as standard of care.

## Background

Glycogen storage disease type II (GSD II, OMIM # 232300), Pompe disease, is an autosomal recessive myopathy caused by lysosomal acid α-glucosidase (GAA) deficiency, which results in accumulation of glycogen mostly in muscle cells. Pompe disease has a broad phenotypical spectrum. The late onset form presents with progressive skeletal muscle weakness with a limb girdle pattern and respiratory insufficiency usually without cardiac involvement [1]. Enzyme replacement therapy (ERT, alglucosidase alfa) results in improved survival, muscle strength and function and stabilization of lung function [2–5]. However, ERT is unable to reverse all muscle damage and the disease remains progressive over time [1, 6].

In addition to ERT exercise training programs tend to be an effective therapeutic intervention in Pompe disease, improving endurance, muscle strength, physical functioning and also fatigue and pain on the short-term [7–11]. One exercise training program combined with high-protein nutrition with a mean duration of 4.5 years in 26 moderate to good compliant adult onset Pompe patients without ERT showed a slower deterioration of muscle function compared to a group of patients not adhering to this program [7]. However, in this study control on adherence and standard follow-up was not always achieved. Other long-term follow-up of healthy physical activity or exercise training programs, in Pompe patients treated with ERT, have not been published thus far.

In general, upholding an active physical lifestyle is a common health advise, as an inactive lifestyle leads to a higher risk of developing non-communicable diseases and has major negative influence on life expectancy and mental health, all of which lead to the rise of global public healthcare costs [12–16]. Therefore, according to the 2010 physical activity norm of the World Health Organization (WHO) healthy adults of age ≥ 18 years should perform at least 150 min of moderate or 75 min of vigorous aerobic physical activity or a combination of both weekly with twice a week bone and muscle strengthening exercises. Moderate activity is defined as activity performed at 3–5.9 times the intensity at rest and vigorous activity is intensity at six or more times the intensity at rest [17]. In both activities heart rate and breathing frequency rise. Moderate activity is for example a brisk walk and riding a bike. Vigorous activity is for example jogging or running. Bone and muscle strengthening activities are recommended for major muscle groups (arms, legs and trunk), examples are lifting weights and heavy gardening [18].

For patients with Pompe disease a long-term active physical lifestyle could also have a positive impact by upholding patients’endurance and muscle strength, while osteoporosis and overweight might be adverted. Furthermore, as in the general population the risk of developing non-communicable diseases might decrease.

Therefore, the aim of this retrospective cohort study was to explore effects of long-term healthy physical activity according to the 2010 WHO norm and to upholding a personalized exercise training program in Pompe disease, by measuring endurance, muscle strength and muscle function. Participants of a former exercise training study performed in 2011 at our center [10] and comparable control patients (fulfilling inclusion criteria of the former study in 2011) were invited to participate. Included patients who in the past 10 years, based on interviews, met healthy physical activity according to the 2010 WHO norm, were compared to patients not meeting this norm on endurance, muscle strength and muscle function. Active patients of the 2011 training cohort who continued the program were also compared to active control patients.

## Materials and methods

### Study design and patients

For this retrospective single center cohort study all patients were recruited at the Center for Lysosomal and Metabolic Diseases (CLMD) at Erasmus University Medical Center, a Dutch international expert and national referral center for patients with Pompe disease. All known patients with Pompe disease in the Netherlands are longitudinally monitored at our outpatient clinic to observe disease progression. Twenty-three patients who took part in a personalized exercise training program in 2011 were approached to participate in this follow-up study. Details of the exercise training program have previously been published [10]. In short, in 2011 participants engaged in a 12 weeks exercise training program focused on endurance, muscle strength and core stability for up to three times a week during 1–1.5 h sessions. The program was executed at local fitness centers under supervision of physical therapists [10].

Inclusion criteria of the former exercise training study in 2011 were: patients were at least 18 years of age and mildly affected (no use of walking or ventilatory devices). Retrospectively, we selected control patients who fulfilled these criteria in 2011 but did not participate in the former exercise training program. This control group had to be treated with at least 7 years of ERT (alglucosidase alfa, 20 mg/kg every other week) at the time of inclusion in the present study comparable to the 2011 training cohort. Patients with concomitant diseases limiting participation in physical activity were not approached. This study was approved by the Ethical Committee at the Erasmus University Medical Center (NL16769.078.07, V17, Amendment 13). Informed consent was obtained from all patients. This trial was also registered at https://trialsearch.who.int/ as Trial NL8508.

### Assessments

Assessments were performed on two separate days. We aimed to perform the two assessment days within 1 month. The following assessments were performed.

#### Interview

Patients of the former 2011 training cohort were interviewed about whether or not they continued training according to the exercise training study in the last ± 10 years, reasons for quitting the program and about their current training regime if any. Furthermore, all study participants (former training cohort and controls) were interviewed about their physical activity in the past ± 10 years to the best of their memory. They were asked to describe whether they performed sports or visited the physical therapist and if so what the training consisted of, how often it was performed and whether they engaged in walking and/or cycling in their daily life activities. Participants were also asked to elaborate on whether they considered fulfilling 150 min of weekly moderate physical activity over the past 10 years. Based on these interviews, current study participants were classified in a physically active and an inactive group according to the 2010 WHO norm.

#### Endurance

Endurance was measured by the peak volume of oxygen uptake (VO2peak, in ml/min/kg) and maximal workload (Wmax, in Watt; Watt/kg) during a maximal incremental cardiopulmonary exercise test (CPET) on a cycle ergometer (Ergoselect 200P, Ergoline GmbH, Bitz, Germany) using a RAMP-protocol as performed and described previously [10]. The test was considered to be maximal when one of the following criteria was met: heart rate reserve < 20 beats per minute, respiratory exchange ratio  > 1.1 or VO2 stabilized despite increased workload. Aerobic capacity (VO2peak) was also evaluated as a percentage compared to reference values [19]. Heartrate, ventilator parameters (VO2, VCO2, breathing frequency, minute ventilation, respiratory exchange ratio) and blood pressure were continuously monitored during the test (Sentry Suite Software, version 3.20).

As a second measure of endurance walking distance on the 6 min walking test (6MWT) was evaluated according to the American Thoracic Society guidelines and presented in meters [20].

#### Muscle strength

Muscle strength testing was performed of muscle groups as described previously [3, 10]. Assessed muscle groups were neck extensors and flexors, pectoralis muscles, shoulder abductors, adductors and exorotators, elbow flexors and extensors, hip flexors, abductors and extensors and knee flexors and extensors. Muscle strength was determined qualitatively by manual testing and scored according to the Medical Research Council (MRC), scores ranging from 0 to 5 [21]. Sum scores were presented as the percentage of the maximum possible score. Quantitatively muscle strength was determined by a hand held dynamometer (HHD) (Citec dynamometer, CT3002/30, CIT Technics Haren, Groningen, Netherlands). HHD values (in Newton) of all tested muscle groups were expressed as a percentage of the median reference value [22], these values were then averaged to a sum score as described previously [3]. When three or more values were missing, no MRC or HHD sum scores were calculated.

#### Muscle function

Functional activity was assessed by the Quick Motor Function Test (QMFT) specifically developed for Pompe disease [23] and presented as a sum score. The test contains 16 items with scores ranging from 0 (not able to perform) to 4 (can perform without effort) examining functional performance of daily life activities, with a maximum score of 64. When three or more values were missing no sum score calculation was made.

#### Lung function

Forced vital capacity in upright and supine position was measured using spirometry (Vyntus One, Vyaire, Wurzburg, Germany) and performed according to the American Thoracic Society Guidelines [24]. This measurement was performed as standard of care where patients wore a nose clip and three repeated flow volume curves were made using the value of the best effort. These values were compared to reference values and presented as percentage predicted [25].

#### Physical activity score

Patients wore an activity monitor (ActiGraph wGT3X-BT, Pensacola Florida) during ± 3 days (preferably 2 week days and one weekend day between the two assessment days). This provided an assessment of the extent of physical activity during waking hours in order to evaluate if this was in line with the activity reported in the interviews and functioned as a verification for the physical activity classification. Activity was presented as a mean of total counts per hour. The activity monitor is able to count the activity in three axes: the sum of all activity counts in the three axes divided by the total wear time resulted in the counts per hour. Data were processed in ActiLife software (version 6 13.4, ActiGraph, Pensacola Florida).

#### Other

Patients’ height and weight were measured and the body mass index was calculated in kg/m^2^ [26].

### Statistical analysis

As sample size was small non parametric tests were used. The differences in the outcomes were compared by the Mann–Whitney U test for unpaired groups. Dichotomous outcomes were compared by the Pearson Chi-square test. Significance level was set at *p* < 0.05. Statistical analyses were performed using SPSS for Windows (IBM SPSS Statistics Version 28.0.1.0, Armonk, NY: IBM Corp).

## Results

### Patients

**Fig. 1:**
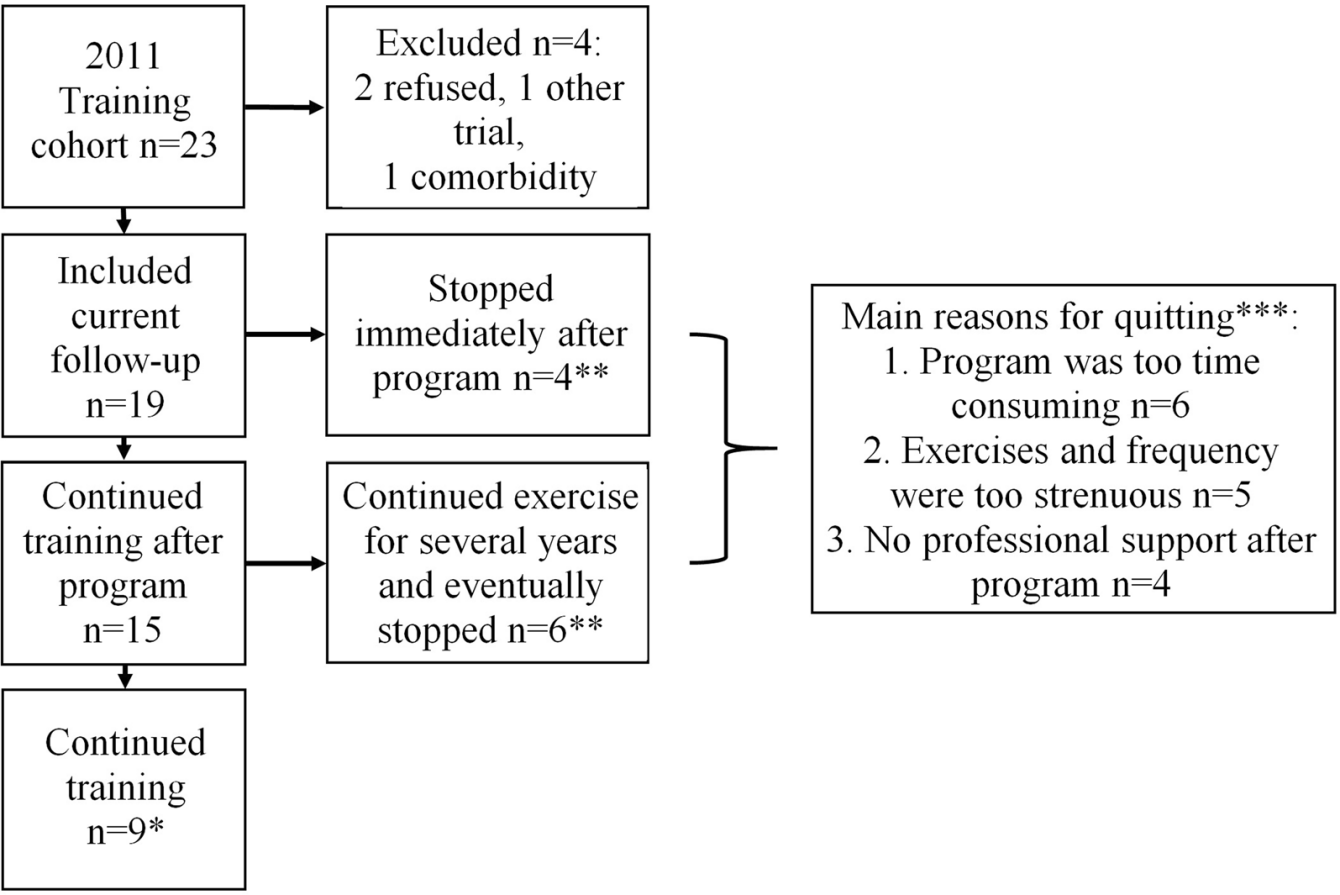
Flowchart 2011 Training cohort. *Patients meet criteria of healthy physical activity according to the 2010 WHO norm. **Patients do not meet the criteria of healthy physical activity according to the 2010 WHO norm. ***Patients gave more than one answer

At the start of the study, in September 2020, a total of 157 late onset Pompe patients were followed at our center. Twenty-three of these patients were former 2011 training cohort participants and 19 of them agreed to participate in the present study. Two refused to participate, one had an interfering comorbidity and one already participated in another trial (Fig. 1). Of the remaining 134 patients 16 patients who did not participate in the former exercise training study were eligible as control patients for the current study according to the previously mentioned selection criteria (no walking aids or ventilatory devices and ≥ 18 years of age in 2011; currently at least 7 years of ERT). From these 16 eligible patients 10 agreed to participate. Reasons for not participating were travel distance, the study being strenuous (two assessment days) and anxiety of getting infected with the corona virus as the study started in a period of an emerging covid-19 pandemic. Therefore a total of 29 participants were included in this study.

We aimed to perform the two assessment days within 1 month, but covid-19 pandemic restrictions interfered with the visits of 14 patients. Participants visited the outpatient clinic within a median interval of two months (range: 1–10). Assessments were performed from 3rd September of 2020 until 14th April of 2022.

### Interview

In the 2011 training cohort (n = 19) nine patients continued training according to the former exercise training program two to three times a week for the whole 10 years up to the moment of inclusion in this study. Four patients stopped exercising according to the program immediately after the end of the study in 2011 and six patients eventually quit after a mean of 2.3 years (range: 1–4). Reasons for quitting the program were multiple: the program being too time consuming to combine with work or caring duties (n = 6), the program being physically too strenuous (n = 5) and lack of professional support at local gyms after the end of the program (n = 4) (Fig. 1).

**Fig. 2:**
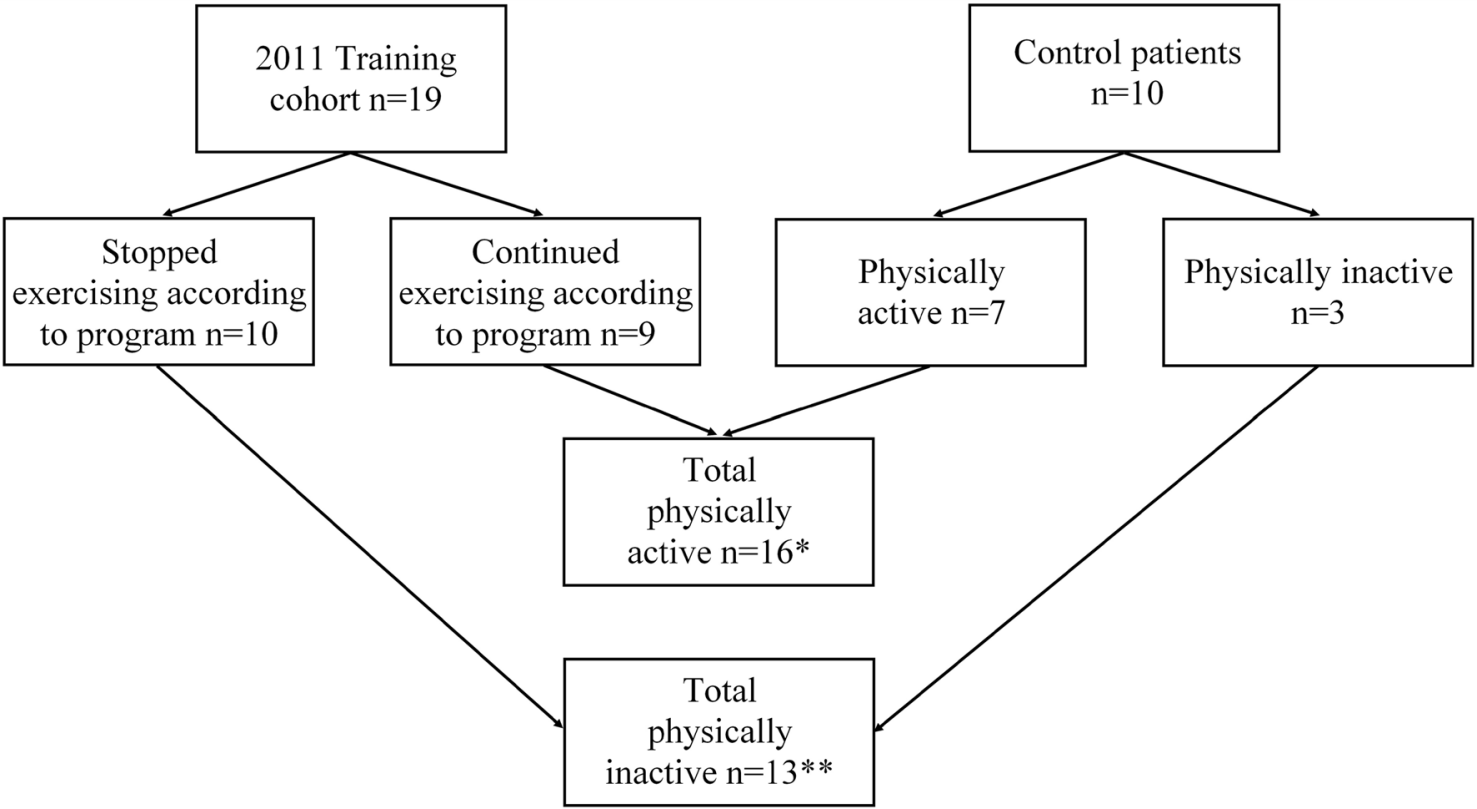
Flowchart physical activity in the past 10 years—2011 training cohort and control patients. *Patients meet the criteria of healthy physical activity according to the 2010 WHO norm. **Patients do not meet the criteria of healthy physical activity according to the 2010 WHO norm

After the interviews of all 29 included patients, we concluded that 16 patients were physically active and 13 patients physically inactive, according to the 2010 WHO norm, in the past 10 years. The physically inactive group mentioned multiple reasons for the inactivity: lack of time (n = 8/13) (mostly when patients were still working), physical limitations (n = 8/13) (too little energy after a long day, fatigue and myalgia after exercise or doubting the ability to perform various exercises), lack of motivation (n = 4/13) (disliking exercise), travel distance to the gym (n = 1/13) and lack of professional support at the gym (n = 1/13) (Fig. 2).

### 2011 Training cohort versus comparable control patients (Tables 1, 2)

**Table 1:** Patient characteristics 2011 training cohort vs. comparable control patients

	2011 Training cohort (n = 19)	Control patients (n = 10)	*p* value
Age (y)	52.0 [42.0;75.0]	48.5 [36.0;74.0]	.261
Male sex n (%)	10 (53%)	7 (70%)	.367
BMI	23.8 [20.0;34.9]	25.6 [20.6;28.3]	.955
Disease duration (y)	17.0 [12.0;41.0]	14.0 [7.0;29.0]	.232
ERT duration (y)	13.0 [11.0;15.0]	10.5 [7.0;15.0]	.058
Ambulation aid n (%)	6 (32%)	3 (30%)	.930
	4 walking stick	2 rolling walker	
	2 partial wheelchair	1 partial wheelchair	
Ventilator support n (%)*	6 (32%)	3 (30%)	.930

All values, if not stated otherwise, are presented as a median with a minimum and maximum range

*BMI* Body mass index, *ERT* enzyme replacement therapy

*Non-invasive overnight ventilator support; Chi-square test for proportions (Asymptotic significance (2 sided)) and Mann–Whitney U test (Exact significance 2-tailed) for continuous unpaired data are used

**Table 2:** Outcome measures 2011 training cohort vs. comparable control patients

	2011 Training cohort (n = 19)	Control patients (n = 10)	*p* value
FVC upright (% of normal)	75 [43;100]	59 [52;89]a	.290
FVC supine (% of normal)	48 [17;84]	34 [23;79]a	.475
VO2peak (ml/min/kg)	17.3 [10.0;35.6]b	18.7 [12.9;29.8]c	.679
VO2peak (% of normal)	64 [36;116]b	59.5 [38;95]c	.466
Wmax (Watt)	84 [26;226]b	89.5 [41;162]c	.966
Wmax/kg (Watt/kg)	1.1 [0.4;2.9]b	1.1 [0.6;1.8]c	.831
6MWT (m)	402.0 [289.0;601.0]d	362.0 [225.0;584.0]a	.329
MRC sum score (% of normal)	83.1 [69.2;98.5]	83.3 [65.0;97.7]a	.933
HHD sum score (% of normal)	82.6 [40.4;96.7]e	90.0 [44.1;97.7]c	.683
QMFT sum score	35 [19;62]	42 [23;60]a	.856
ActiGraph activity score (counts/hour)	36834.8 [12047.3;97689.5]e	38492.4 [17206.7;49131.0]a	.781

All values, if not stated otherwise, are presented as a median with a minimum and maximum range; a n = 9, b n = 17, c n = 8, d n = 16, e n = 18, missings due to inadequate technical performance or not performed due to logistic reasons; Mann–Whitney U test (Exact significance 2-tailed) is used

*FVC* Forced vital capacity, *VO2peak* Peak oxygen uptake, *Wmax* Maximum workload, *6MWT* 6 Minute Walking Test (maximum walking distance in 6 min), *MRC* Medical Research Council (manual muscle strength test), *HHD* Hand Held Dynamometer (quantitative muscle strength test), *QMFT* Quick Motor Function Test

As control patients were selected on their comparability with the 2011 training cohort no significant differences were found in all patient characteristics. Patients in the 2011 training cohort had a median age of 52 years versus 48.5 years in control patients (*p* = 0.261). Disease duration (from diagnosis) was respectively 17 and 14 years (*p* = 0.232) and treatment with ERT 13 and 10.5 years (*p* = 0.058) and no differences were seen between sex, BMI and ambulation or ventilator aid in these groups (Table 1). Exploring all outcomes (FVC, VO2peak, Wmax, 6MWT, MRC, HHD, QMFT and activity score) between these two groups no significant differences existed (Table 2).

### Physically active versus inactive patients (2010 WHO norm) (Tables 2, 3)

**Table 3:** Patient characteristics physically active vs. inactive patients (2010 WHO norm)

	Physically active (n = 16)	Physically inactive (n = 13)	*p* value
Age (y)	52.0 [39.0;73.0]	50.0 [36.0;75.0]	.821
Male sex n (%)	11 (69%)	6 (46%)	.219
BMI	24.8 [22.0;33.0]	26.7 [20.6;34.9]	.722
Disease duration (y)	16.0 [8.0;31.0]	17.0 [7.0;41.0]	.523
ERT duration (y)	12.5 [7.0;15.0]	12.0 [7.0;14.0]	.533
Ambulation aid n (%)	4 (25%)	5 (38%)	.436
	2 walking stick	2 walking stick	
	1 rolling walker	1 rolling walker	
	1 partial wheelchair	2 partial wheelchair	
Ventilator support n (%)*	4 (25%)	5 (38%)	.436

All values, if not stated otherwise, are presented as a median with a minimum and maximum range

*BMI* Body mass index, *ERT* enzyme replacement therapy

*Non-invasive overnight ventilator support; Chi-square test for proportions (Asymptotic significance (2 sided)) and Mann–Whitney U test (Exact significance 2-tailed) for continuous unpaired data are used

Patient characteristics were not significantly different between the physically active and inactive patients. There tended to be more male patients (69 vs. 46%, *p* = 0.219) and a lower median BMI (24.8 vs. 26.7 kg/m^2^, *p* = 0.722) in the active group (Table 3). Median of VO2peak/kg (19.4 vs. 16.6 ml/min/kg, *p* = 0.026), Wmax (107 vs. 54 Watt, *p* = 0.007),Wmax/kg (1.4 vs. 0.7 Watt/kg, *p* = 0.003), HHD sum score (92.5 vs. 80.3%, *p* = 0.027), QMFT sum score (42.5 vs. 29.5 *p* = 0.005) and activity score (40483.1 vs. 31209.6 counts/hour, *p* = 0.001) were significantly higher in the active compared to the inactive group. The median of FVC upright and supine, VO2peak (% of normal), 6MWT and MRC sum score tended to be higher in the active group (*p* > 0.05) (Table 4). There were two partial wheelchair users in the physically inactive versus one wheelchair user in the active group. Patients in a wheelchair are more affected by the disease, however the significant difference in the outcomes remained when wheelchair patients (n = 1 in the active and n = 2 in the physically inactive group) were omitted from analysis (data not shown).

**Table 4:** Outcome measures physically active vs. inactive patients (2010 WHO norm)

	Physically active (n = 16)	Physically inactive (n = 13)	*p* value
FVC upright (% of normal)	81 [54;100]	65.5 [43;85]a	.107
FVC supine (% of normal)	52 [26;84]	41 [17;58]a	.124
VO2peak (ml/min/kg)	19.4 [12.9;35.6]b	16.6 [10.0;21.4]c	.026
VO2peak (% of normal)	65 [36;116]b	58 [38;108]c	.798
Wmax (Watt)	107 [41;226]b	54 [26;91]c	.007
Wmax/kg (Watt/kg)	1.4 [0.6;2.9]b	0.7 [0.4;1.3]c	.003
6MWT (m)	453.0 [225.0;601.0]d	365.0 [237.0;544.0]e	.338
MRC sum score (% of normal)	85.8 [69.2;98.5]	80.0 [65.0;94.6]a	.142
HHD sum score (% of normal)	92.5 [60.9;97.7]d	80.3 [40.4;93.4]c	.027
QMFT sum score	42.5 [29;62]	29.5 [19;47]a	.005
ActiGraph activity score (counts/hour)	40483.1 [20768.3;97689.5]	31209.6 [12047.3;42385.2]c	.001

All values, if not stated otherwise, are presented as a median with a minimum and maximum range; a n = 12, b n = 14, c n = 11, d n = 15, e n = 10, missings due to inadequate technical performance or not performed due to logistic reasons; Mann–Whitney U test (Exact significance 2-tailed) is used

*FVC* Forced vital capacity, *VO2peak* Peak Oxygen Uptake, *Wmax* Maximum Workload, *6MWT* 6 Minute Walking Test (maximum walking distance in 6 min), *MRC* Medical Research Council (manual muscle strength test), *HHD* Hand Held Dynamometer (quantitative muscle strength test), *QMFT* Quick Motor Function Test

### Physically active 2011 training cohort (continued training) versus active controls (Tables 5, 6)

**Table 5:** Patient characteristics physically active 2011 training cohort (continued training) vs. physically active controls

	Physically active 2011 training cohort (n = 9)	Physically active controls (n = 7)	*p* value
Age (y)	52.0 [45.0;66.0]	50.0 [39.0;73.0]	.364
Male sex n (%)	6 (67%)	5 (71%)	.838
BMI	23.7 [20.0;33.0]	25.9 [23.1;28.3]	.201
Disease duration (y)	17.0 [13.0;31.0]	13.0 [8.0;20.0]	.086
ERT duration (y)	13.0 [11.0;15.0]	11.0 [7.0;15.0]	.281
Ambulation aid n (%)	2 (22%)	2 (29%)	.771
	2 walking stick	1 rolling walker	
		1 partial wheelchair	
Ventilator support n (%)*	2 (22%)	2 (29%)	.771

All values, if not stated otherwise, are presented as a median with a minimum and maximum range

*Non-invasive overnight ventilator support; Chi-square test for proportions (Asymptotic significance (2 sided)) and Mann–Whitney U test (Exact significance 2-tailed) for continuous unpaired data are used

*BMI* Body mass index, *ERT* enzyme replacement therapy

**Table 6:** Outcome measures physically active 2011 training cohort (continued training) vs. physically active controls

	Physically active 2011 training cohort (n = 9)	Physically active controls (n = 7)	*p* value
FVC upright (% of normal)	82 [55;100]	59 [54;89]	.133
FVC supine (% of normal)	56 [32;84]	34 [26;79]	.262
VO2peak (ml/min/kg)	21.2 [14.6;35.6]a	19.4 [12.9;29.8]b	.825
VO2peak (% of normal)	67 [36;116]a	59.5 [40;89]b	.395
Wmax (Watt)	107 [47;226]a	108 [41;162]b	.950
Wmax/kg (Watt/kg)	1.5 [0.7;2.9]a	1.3 [0.6;1.8]b	.640
6MWT (m)	460.5 [289.0;601.0]a	366.0 [225.0;584.0]	.613
MRC sum score (% of normal)	87.7 [69.2;98.5]	83.8 [74.4;97.7]	.939
HHD sum score (% of normal)	88.4 [60.9;96.7]	92.7 [64.2;97.7]b	.607
QMFT sum score	37 [31;62]	43 [29;60]	1.000
ActiGraph activity score (counts/hour)	47150.1 [20768.3;97689.5]	39771.1 [34180.0;49131.0]	.252

All values, if not stated otherwise, are presented as a median with a minimum and maximum range; a n = 8, b n = 6, missings due to inadequate technical performance or not performed due to logistic reasons; Mann–Whitney U test (Exact significance 2-tailed) is used

*FVC* Forced Vital Capacity, *VO2peak* Peak Oxygen Uptake, *Wmax* Maximum Workload, *6MWT* 6 Minute Walking Test (maximum walking distance in 6 min), *MRC* Medical Research Council (manual muscle strength test), *HHD* Hand Held Dynamometer (quantitative muscle strength test), *QMFT* Quick Motor Function Test

None of the patient characteristics were different between these groups (*p* > 0.05). Patients who continued training tended to have a longer median disease duration (from diagnosis) (17 vs. 13 years, *p* = 0.086) (Table 5).

The median of eight of the outcomes tended to be higher in the former training cohort who continued training compared to active controls, not reaching significance. There was a trend of a higher median of FVC upright (82 vs. 59%, *p* = 0.133) and supine (56 vs. 34%, *p* = 0.262), endurance (VO2peak/kg, 21.2 vs. 19.4 ml/min/kg, *p* = 0.825; VO2peak % normal, 67 vs 59.5%, *p* = 0.395; Wmax/kg, 1.5 vs 1.3 Watt/kg, *p* = 0.640), 6MWT (460.5 vs. 366 m, *p* = 0.613), MRC sum score (87.7 vs. 83.8%, *p* = 0.939) and activity score (47150.1 vs. 39771.1 counts/hour, *p* = 0.252) in the former training cohort who continued training. The HHD sum score was 88.4 vs. 92.7% (*p* = 0.607), the QMFT sum score 37 vs. 43, (*p* = 1.000) and Wmax 107 vs. 108 Watt (*p* = 0.950) respectively in the former training cohort who continued training and the active controls (Table 6).

## Discussion

This study investigated long-term effects of healthy physical activity according to the 2010 WHO norm and the former personalized exercise training program in adults with late onset Pompe disease. Endurance, muscle strength and muscle function were significantly higher in the physically active compared to the inactive group. The majority of the outcomes tended to be higher in the 2011 training cohort still following the former exercise training program compared to active control patients who did not participate in the former exercise training study, even though the active 2011 training cohort tended to have a longer disease duration. This study thus supports the positive effect of physical activity on delaying disease progression in Pompe patients. Furthermore, the difference in the median of VO2peak between active and inactive patients was 2.8 ml/min/kg, which is a highly relevant difference as in the general population 1 ml/min/kg higher VO2peak is associated with a 9% relative risk reduction in all-cause mortality [27]. A 2.8 ml/min/kg difference in VO2peak could be even more important for the wellbeing of patients with Pompe disease compared to healthy controls as their exercise tolerance (VO2peak) is already reduced compared to healthy age-matched sedentary individuals [28].

We considered the difference between the active and inactive group representing a difference in disease severity. However, patient characteristics including use of ambulation and ventilatory aids were comparable. Furthermore, when omitting the wheelchair users from the analysis, being the most severely affected participants, no changes in the significance occurred between the active and inactive groups. Besides, in the interviews, physically inactive patients not only mentioned physical but also time limitation as the reason for their inactivity. Therefore, it is likely that the differences in the outcomes are indeed caused by the difference in physical activity. To correct for disease severity as a confounder a randomized trial with a long duration time in which no other therapies are changed should be performed. However, such a trial is not feasible as we cannot deny patients exercise and new therapies which have beneficial health effects.

We expected the outcomes of the former 2011 training cohort who continued training to be superior to physically active control patients (according to the WHO norm). We expected this because the former program was more vigorous, personalized, patients were initially instructed to perform exercises correctly and they tended to have a higher median activity score according to the activity monitor. Although the majority of the outcomes tended to be higher in the active 2011 training cohort, due to small patient numbers included in our current study, no significance was reached. Moreover, after the success of the former exercise training program in 2011 the main pillars of the program: endurance, strength and core stability were implemented as standard clinical practice in our center. Local physical therapists who treat the patients are advised by physical therapists from our hospital to implement comparable training programs. Therefore, the active controls were already guided by their therapists according the principles of the former study, which can explain the small outcome differences between these groups.

In general, healthy physical activity could lower the risk of occurrence of non-communicable diseases such as cardiovascular disease and diabetes [12, 15]. This is of major relevance in Pompe disease, because due to lower muscle mass and consequently decreased resting energy expenditure adult Pompe patients have an increased risk of developing sarcopenic obesity [29, 30]. Sarcopenic obesity is associated with the metabolic syndrome and an increased cardiovascular morbidity risk, which also may already be increased in Pompe disease [31]. In our study the median BMI of the physically inactive patients tended to be higher (not significant) compared to active patients. With regular physical activity patients could prevent obesity and maintain a healthy body weight. This may be particularly interesting for patients with Pompe disease as ERT is dosed per kg body weight and prevention of obesity in Pompe patients may thus also lower health care costs. Besides, reduced skeletal muscle traction forces due to reduced mobility could eventually lead to decline in bone mineral density and osteoporosis posing a risk factor for fractures, already shown to occur in Pompe disease [32–35]. Physical activity on the long-term may thus also help Pompe patients to prevent osteoporosis and risk of fractures. We therefore conclude that physical activity is of great value for the overall wellbeing of patients with Pompe disease and therefore should be implemented as an important element of standard of care.

In the future more research is needed on suitable training regimes for more severely affected Pompe patients during their disease course as patients also mentioned physical limitations as a holdback for physical activity. These patients may be motivated for regular exercise when provided personal exercise adjustments alongside professional support.

The strengths of this study are the long follow-up time and interviews with patients on their motivations for upholding physical activity which have not been explored previously. Limitations of this study are the small sample size and the retrospective nature of the study which could have led to a memory bias. Due to the retrospective nature it is difficult to determine whether patients were indeed as active in the past 10 years as mentioned by them. We also considered the possibility that the most active patients wanted to participate in this study, however, a brief interview of the six eligible controls who did not want to participate, learned that five of them were also physically active according to the 2010 WHO norm. Due to a small sample size and given the explorative nature of this study concerning a rare disease no correction for multiple testing was performed. At last, patients’ mental and physical wellbeing could be influenced by the covid-19 pandemic, which could have influenced the fitness of our study population.

Of note, in 2020 an update on the WHO norm has been published, in which the minimum criterion is changed to a range of 150–300 min of moderate intensity or 75–150 min of vigorous intensity of physical activity a week [36].

## Conclusion

Our study shows that physically active Pompe patients, upholding the 2010 WHO healthy physical activity norm or the more vigorous exercise training program by van den Berg et al on the long-term, experience benefits on endurance, muscle strength and function. Thus, patients should be motivated to stay physically active according to the 2010 WHO norm at a minimal to delay disease deterioration and prevent comorbidities. When necessary, adequate professional support and exercise adjustments should be implemented in the training sessions to meet patients’ physical abilities and to uphold exercise motivation.

## Data Availability

The datasets generated and analyzed during the current study are anonymised and available as open data via the Erasmus University Rotterdam online data repository: https://pure.eur.nl/en/publications/long-term-benefits-of-physical-activity-in-adult-patients-with-la. The interviews taken during the current study are not publicly available due to specific patient related information, but are available from the corresponding author on reasonable request.
